# Perceptions of facilitators, barriers and solutions when preparing to implement a home visiting program in Sweden: a mixed-methods study

**DOI:** 10.3389/frhs.2024.1335559

**Published:** 2024-03-18

**Authors:** Julie S. Lundgren, Åsa Nilses, Ebba-Lisa Eckerdal, Susanne Bernhardsson

**Affiliations:** ^1^ Region Västra Götaland, Center for Progress in Children’s Mental Health, Child and Youth Health Specialty Services, Regional Healthcare, Gothenburg, Sweden; ^2^ Region Västra Götaland, Research, Education, Development and Innovation Primary Health Care, Vänersborg, Sweden; ^3^ Department of Health and Rehabilitation, The Sahlgrenska Academy, Institute of Neuroscience and Physiology, University of Gothenburg, Gothenburg, Sweden

**Keywords:** implementation science, extended home visits, parenting, child health services, sustainable development, social work

## Abstract

**Background:**

Although there is growing awareness that early childhood development programs are important for a sustainable society, there is a knowledge gap about how to implement such programs. Successful implementation requires attention to implementation drivers (competency, organization, and leadership) during all phases of the implementation. The purpose of this study was to describe cross-sectoral operational workgroups’ perceptions of facilitators, barriers and solutions related to implementation drivers in the preparationphase of implementing an evidence-based early childhood home visiting program.

**Methods:**

Quantitative and qualitative data were collected from twenty-four participants, divided into 5 groups, during implementation planning workshops. The workshops were guided by a structured method informed by the principles of Motivational Interviewing and within a framework of implementation drivers. Groups sorted cards with statements representing implementation drivers according to perceptions of facilitators and barriers, and percentages were calculated for each type of implementation determinant, for each type of driver. The groups discussed their card sorting and wrote action plans to address barriers, yielding documentation that was analyzed using deductive qualitative content analysis.

**Results:**

A mixed-methods analysis resulted identification of facilitators, barriers, unknowns and solutions in two to three subcategories under each main category of implementation driver. A competent and confident workforce, and enthusiasm and commitment were key facilitators. Key barriers were unclear roles and responsibilities, and insufficient articulation of local vision and goals. Many factors were described as yet unknown. Specific solutions were generated to support the implementation.

**Conclusions:**

Our study furthers the scientific understanding of how to take evidence-based early childhood programs from research to practice within an implementation drivers framework. Facilitators, barriers and solutions in key areas during the preparation phase were identified with the help of a novel tool. The results provide useful knowledge for decision makers and organizations preparing similar initiatives in communities striving to attain sustainable development goals.

## 1 Introduction

Creating an equitable society where all citizens have access to and participate in services that promote the conditions for health and well-being is embedded in the United Nations’ Sustainable Development Goals (SDGs) (1). The World Health Organization Commission on Social Determinants of Health (2) states that health inequities can be avoided by targeting malleable factors affecting human growth conditions. Parenting is one such factor that can serve a powerful protective function for youth growing up in marginalized communities (3). Post-natal home visiting programs have short- and long-term positive effects on conditions important for children's early development (4–6). There is evidence for the effectiveness of home visiting in infancy and early childhood to families in socio-economically disadvantaged areas, and an expanded number of visits can improve children's development and health (7). Home visiting programs seek to improve parents’ knowledge and skills, and also target contextual factors affecting families living in disadvantage, such as economic independence, social inclusion, and networking (5).

A Swedish-developed infant home visiting program (8), created and evaluated in Rinkeby, a marginalized district in the city of Stockholm, has the promise for contributing to the attainment of SDG 3 (Good health and well-being), SDG 10 (Reduced inequalities) and SDG 17 (Partnerships for the goals) (1, 9). The program, Rinkeby extended home visiting (REHV), involves cross-sectoral collaboration between child health clinics (CHC) in the health sector and social services at the municipal level. A child health nurse and a family support social worker carry out six home visits during the child's first 15 months of life to families in vulnerable areas who have had their first child, or first child born in Sweden. The program is an extension of the national universal healthcare program offered at CHCs, in which two home visits delivered by nurses are offered as part of usual care. The core REHV program components correspond both with those shown in previous research to be effective and with the Nurturing Care framework for social sustainability proposed by the World Health Organization (9, 10). Evaluations have demonstrated good effects on both child and parental well-being compared with families receiving standard care (11–13). Success with multiple implementation components have been reported, such as the perceived appropriateness, acceptance, and uptake of the method in routine care (12). Implementations in other areas of Sweden have shown that the program is perceived as valuable and feasible in new contexts (11, 14, 15).

To attain positive outcomes with early childhood programs, careful attention to the implementation process is needed (16). Many organizations are aware of the need for nurturing care programs but lack knowledge about how to implement them (17). Previous research on implementation of early childhood interventions indicates the usefulness of applying models for implementation drivers and phases (18). Implementation drivers are basic organizational conditions for an effective and sustainable implementation in three categories: Competency drivers, Organizational drivers, and Leadership drivers (19, 20). Competency drivers support professional development and include selection, training, and supervision. Organizational drivers provide structures and systems such as administrative guidelines, allocation of resources, and data systems for fidelity and outcome evaluation. Leadership drivers encompass the technical and flexible leadership required to manage the impact of change during implementation. Different implementation phases call for different activities, roles, and resources to support implementation drivers. Four phases of implementation have been proposed: exploration, preparation, implementation, and sustainment (21) and activities to secure implementation drivers need to be continually monitored across all phases of an implementation (20).

Organizational readiness for implementing a new method entails preparing a strategic and motivational organizational climate to support implementation (22). The provision of pre-implementation readiness support is associated with better sustainability, but concrete materials to facilitate readiness are often lacking in evidence-based methods (23). Key factors facilitating readiness include an established commitment and motivation for implementing the change, leadership style, program consistency with agency vision and goals, management processes and fidelity, organizational stability, a history of successful change, and a culture of professional development (22, 23). In the preparation phase, assessment of facilitators and barriers, consensus building, and problem solving are recommended activities (24). Previous research on the implementation of infant home visiting programs described the importance of investing time and energy in the preparation phase (25). Organizations can vary widely in their capacity for supporting the implementation of change (26). Leadership at different levels, including managers and workgroup leaders, play a pivotal role in securing organizational readiness for implementing change in the preparation phase (27).

The purpose of this study was to describe operational workgroups’ perceptions of facilitators, barriers and solutions related to implementation drivers in the preparation phase of implementing REHV in a new context (Gothenburg). The study addresses a knowledge gap for implementation researchers and practitioners alike about taking nurturing care programs from research to practice and can inform cross-sectoral implementations of other similar early childhood initiatives. The research questions were: What factors are perceived as facilitators and barriers in the preparation phase of the implementation? What solutions (resources, plans or methods) do participants perceive to be needed to create conditions for successful implementation?

## 2 Materials and methods

### 2.1 Study design

This study employed a parallel, convergent, mixed-methods approach integrating quantitative and qualitative data (28, 29). The mixed methods approach was applied during data collection, analysis, and interpretation. Data were collected concurrently and integrated in the analysis, giving equal weight to the two datasets.

### 2.2 Context

#### 2.2.1 Cross-sectoral partnership: family-centered approach

This study took place in the city of Gothenburg, Sweden in the fall of 2018. In Sweden, healthcare is decentralized, with management dispersed at national, regional, and local levels (30). Primary healthcare, including public health and preventive care, is the responsibility of regional government councils. Municipalities are responsible for the local environment of the citizens, for example schools and social welfare services. Gothenburg, the largest municipality in Västra Götaland, is a partner in the World Health Organization's Healthy Cities initiative (31). Healthy cities go hand in hand with the SDGs: “A healthy city is one that puts health, social well-being, equity and sustainable development at the centre of local policies” (31). In 2018, a joint decision was made between the healthcare sector and municipal social services in Västra Götaland to implement the REHV program in areas designated as marginalized in the city of Gothenburg (32). The REHV implementation is an expansion and strengthening of an existing partnership between the Regional Health and Medical Council and the Municipality Council in Gothenburg to promote equitable access to universal child and family services through a family-centered approach (FCA). In the Gothenburg model for FCA, forms for collaboration between midwifery clinics, CHCs, open play schools, and family social services have been established, and within certain districts these functions are co-located in family centers. Each district in the city of Gothenburg has an FCA coordinator co-financed by the FCA initiative (32).

#### 2.2.2 Implementation support

**Figure 1:**
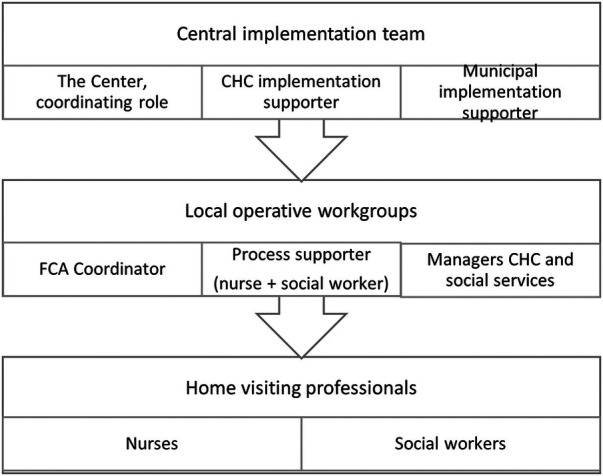
Implementation support in the implementation of the Rinkeby home visiting program in Gothenburg.

A central implementation team and local operational workgroups were assigned responsibility for managing the implementation. The central implementation support team was coordinated by The Center for Progress in Children's Mental Health (the Center), a unit within Region Västra Götaland tasked with supporting and evaluating the implementation of evidence-based programs for children's mental health. Staff at the Center were responsible for coordination of implementation roles and responsibilities, training and supervision of home visitors, planning for fidelity assurance, and outcome evaluations of the implementation. The first, second and third authors work at the Center and conducted this study as part of a larger planning effort to study the REHV implementation in Gothenburg. Local operational workgroups were established to manage local needs and processes, comprising managers of both CHC and social service agencies, FCA coordinators, and process supporters. The latter were nurses and social workers who would be doing home visits and also have responsibilities for facilitating the implementation process. Process supporter was a new role developed for the REHV initiative to facilitate communication of needs between home visiting staff locally and the central implementation support team, and to facilitate data collection for fidelity monitoring and outcomes evaluations. The structure of implementation support is depicted in Figure 1.

### 2.3 Participants

Thirty-one individuals with different roles in the REHV implementation were invited to attend the workshops at an inspiration start-up day. Seven invitees declined participation. Twenty-four (77%) participated in the workshops in which data were collected for this study. Twenty-two of the attendees were members of operational workgroups representing CHC and social service agencies in one of three different communities as depicted in Figure 1. Two persons with other key roles in FCA also participated. All groups were in the preparation phase and members had different roles in the REHV implementation (Table 1). Seventeen (71%) participants submitted demographic information. All but one of the participants who did not submit demographic information were from the CHCs (nurse process supporters and CHC managers). All participants were women. The average length of professional experience was 22 years (SD = 6; range 4–33 years). The participant average age was 46 years (SD = 5.3 years; range 36–57 years).

**Table 1:** Workshop session group compositions.

Group	1	2	3	4	5	Total
Role						
Process supporter—nurse	1	2	1	3	1	8
Process supporter—social worker	1	1		1	1	4
Manager, CHC	1	1	1		1	4
Manager, social services	1		1		1	3
FCA coordinator		1		1	1	3
Other key role in FCA			1	1		2
Total number of participants per group	4	5	4	6	5	24

CHC, child health clinic; FCA, family-centered approach.

### 2.4 Procedure

Study procedures were planned with dual purposes in mind: to support operational workgroups in implementation planning and to study the implementation process. To minimize extra time demands for participants, data collection took place during inspiration start-up day for workgroups preparing to implement the REHV. The operational workgroups participated in a 90-minute workshop in Gothenburg to prepare for their local implementations. The workshops began with a 15-minute PowerPoint presentation held by the first author with a brief description of implementation drivers and phases with particular attention to the preparation phase. Participants were then divided into five groups of 4–6 according to operational workgroup membership in each district. Each group was facilitated by two people affiliated with the Center, one to lead the workshop, giving instructions and asking follow-up questions if clarification was needed, and one to observe and take field notes. Workshop facilitators were bachelor's and master's level social workers, three of whom were female and one male, with previous training in Motivational Interviewing. The facilitators were known to some of the participants from previous implementations, and all participants were aware of their affiliation with The Center and responsibility for coordinating the REHV implementation. The first author served as an observer/documenter in one of the groups.

Data were collected using a tool called IMPLEMENTATION DECK (33). This tool is constructed as a card game that teams of professionals play together, based on Fixsen et al.'s (20) model of implementation drivers and Motivational Interviewing (34). It contains 54 cards with statements that reflect one of the three implementation drivers (18 cards for each driver). This is the first time the tool is used in research. The rationale for selecting IMPLEMENTATION DECK stemmed from previous research highlighting the usefulness of integrating motivational interviewing when implementing evidence-based programs to attain SDGs (35, 36). IMPLEMENTATION DECK is consistent with the recommended core features of group-based alternatives for evaluating organizational readiness (37). Examples of the cards’ statements in the various implementation drivers are shown in Table 2.

**Table 2:** Examples of card statements in IMPLEMENTATION DECK.

Implementation driver	Card statement
Competency drivers	Our method supervisor does not seem to know the method themselves.
	We need training support to be able to use the method.
	Unfortunately, we have chosen the wrong people to be responsible for the method.
	We have staff who have dropped out as method supporters because they have felt inadequate.
Organizational drivers	We have many methods that compete for both time and money.
	We don't get any support from the leadership over us.
	It is not entirely clear who is doing what the implementation process with the method.
	We in the management team may not be open enough with each other when we talk about methods that we work with.
Leadership drivers	Management has difficulty getting employees to work with the method.
	The long-term work is hampered by everything that needs to be addressed “urgently”.
	The method does not fit with our way of working.
	Those who will work with the method have a strenuous job, so we have to be lenient if they sometimes don't comply with the method.

**Figure 2:**
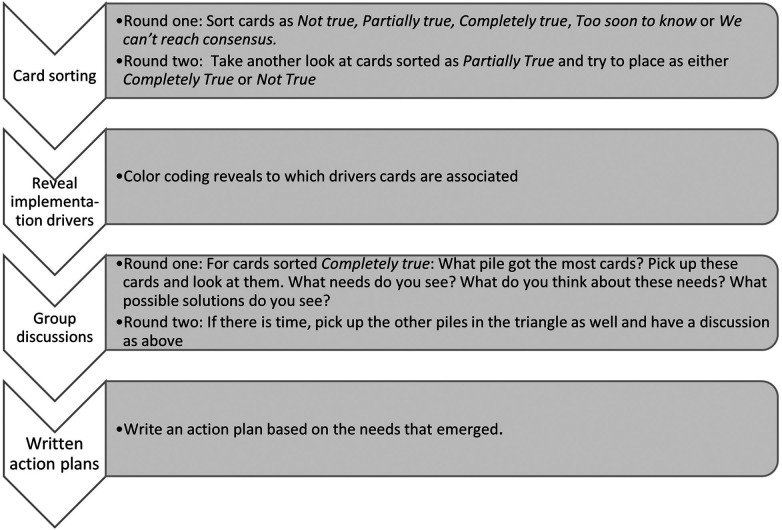
Mixed methods procedural approach.

Figure 2 describes the procedural steps for card sorting and group discussions, and how the two steps built on each other. Step one involved the collection of quantitative data through groups’ sorting of cards. The sorted cards were then used to facilitate the discussions that served as qualitative data. Those responsible for documenting during the workshops were instructed to indicate which cards were sorted into which alternatives, and to capture the group discussions following the card sorting in as much detail as possible. The documentation of discussions in response to card sorting and of written action plans ranged in length between 300 and 1000 words. Longer field notes included documentation during the card sorting task which, although not required, provided somewhat richer material from those groups.

All statements are negatively worded such that the cards sorted into the Completely True pile reflect perceived barriers, and the cards placed in the Not true pile reflect perceived facilitators in the implementation. There was a fifth response option, We can't reach consensus, but none of the groups used this alternative.

### 2.5 Data analysis

**Figure 3:**
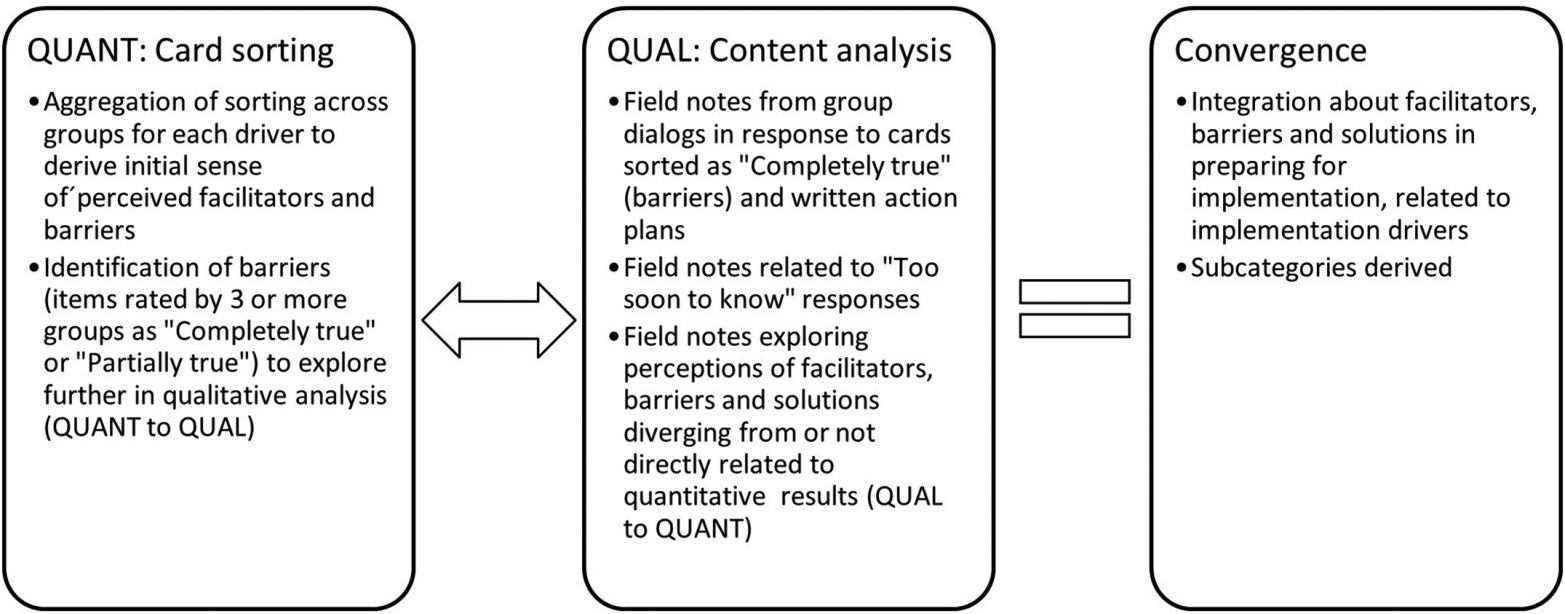
Mixed methods analytical approach.

Using a mixed-methods analytical approach (Figure 3), data were analyzed in several steps. Data sets were summarized independently and then integrated by merging them as described by Creswell and Plano Clark (38). Quantitative results from card sorting informed initial areas of exploration in the content analysis, but the content analysis was not limited to results from the card sorting. Qualitative data also served to compliment, expand and deepen the initial understanding of facilitators, barriers and needed solutions related to implementation drivers.

Quantitative results consist of the total number of cards sorted into each response alternative in each of the three categories of implementation drivers. Because so few cards were sorted as *Partially true* (6 cards, 2%) the decision was made to group these cards as *Completely true*. Next, the number of cards in each response alternative was divided by 90 (18 cards × 5 groups) yielding three summary scores (*Completely true, Not true, and Too soon to know*) for each alternative. Cards sorted as either *Completely* or *Partially true* by 3 or more groups were initially labeled barriers, and these statements guided step one in the qualitative analysis as shown in Figure 3. Cards sorted *Not true* were labeled facilitators. Cards sorted as *Too soon to know* were labeled “Unknowns” and explored further in the qualitative analysis.

The qualitative data were analyzed using deductive content analysis, an approach that is appropriate when analysis has its starting point in a previously established theory or model (39), as is the case with our use of the implementation drivers framework. The analysis was guided by the steps outlined by Graneheim & Lundman (40). The unit of analysis was the entire written material from the workshop session, including written field notes documenting group discussions and action plans. The field notes and action plans were initially analyzed by the first author. The material was read through several times to gain familiarity independent of the card sorting. The second step started with selection of meaning units from the field notes, followed by generation of condensed meaning units and assignment of codes. The codes were labeled as either facilitators, barriers, unknowns or solutions. Facilitators, barriers, unknowns and solutions identified were sorted into the relevant subcategories, which were then consolidated into subcategories. Next, the subcategories were organized into one of the three main categories of Implementation drivers: Competency, Organizational and Leadership. Field notes were read through to identify which of the cards gave rise to the discussions and solutions in the qualitative material. Both the card sorting results and the content analysis of the discussions informed the categorization of the integrated data and labeling of categories and subcategories. A final step involved a back-and-forth movement between raw data and coded material, refining the fit of the raw data. Coding was verified by the second and third authors, who independently of one another checked the coding matrix and compared it with the field notes. This verification led to minor adjustments of a few codes and discrepancies were resolved in consensus. To illustrate the analytical process, an excerpt from the coding matrix is presented in Table 3.

**Table 3:** Excerpt from coding matrix to illustrate the analysis process.

QUANT	QUAL		Integration			
Card sorting	Meaning unit	Condensed meaning unit/code	Barrier (B), Facilitator (F), Unknown (U), Solution (S)	Subcategory		Main category
3 cards relating to the need for training sorted as *Completely True* and/or *Partially true* by all groups	Many strengths in competence, the group has the right competence for the task and the group feels secure.	Right competence, feeling secure	F, S	Confident and competent workforce based on previous experience (F)	Learning together	Competency
	Those experienced providing family support in teams, home visits could start now even without training	Those with experience could start without training	F, S			
	Those afraid to do home visits need support.	Support for those afraid to do home visits	B, S			
4 cards relating to unclear roles sorted as *Completely True* by 4 groups	Roles and responsibilities inadequately defined.	Unclear roles	B, U	Unclear roles and responsibilities in the implementation (B)	Process supporter role	Organization
	It isn't clear for the managers what the process supporters are supposed to do.	Process supporter role unclear for managers	B			
	Clarity about the process supporters, both in the operative group and in relation to staff.	Clarify process supporter role in relation to operative workgroups and staff	B, S			
3 cards relating to staff engagement and motivation sorted *Not True* by all 5 groups	Staff engagement is a facilitator, creates motivation for the implementation.	Staff engagement is a facilitator, creates motivation	F	Professionals’ willingness, engagement, enthusiasm (F)	Inner and outer context	Leadership
	It's a new way of working but everyone is on board and positive to the change.	Everyone on board, positive	F			
	The method is seen as a given.	Method is a given	F, S			

B, barrier; F, facilitator; S, solution; U, unknown.

### 2.6 Ethical considerations

Ethical permission was applied for to the Gothenburg Regional Ethics Review Board. The board returned a decision that the study did not fall under their purview and ethical permission was not required. (Exp. 2018-10-11; 751-18). Participants received oral and written information about the study and were given the opportunity to ask questions. This was done both in advance and again on the day of data collection prior to the workshop, after which the participants provided oral informed consent. Attendance was voluntary and could be withdrawn at any time. Responses to questions were handled confidentially and are presented in such a way that no individual participant can be identified. Groups were assigned numbers to protect the participants’ anonymity.

## 3 Results

Analysis of the quantitative data resulted in a predominance of cards sorted as facilitators. The fact that very few cards were sorted *Partially true* and no cards sorted as *We can't reach consensus* suggests that the groups had relatively coherent views of the implementation drivers. Leadership drivers had the highest proportion of facilitators. Organizational drivers had the highest percentage of barriers and the lowest percentage of *Too soon to know.* The highest percentage of *Too soon to know* cards pertained to Competency drivers. Analysis of the qualitative data and integration of the two datasets resulted in the identification of facilitators, barriers, and unknowns for each implementation driver, grouped into two to three subcategories under each main implementation driver category.

### 3.1 Competency drivers

Table 4 presents a summary of the findings in relation to competency drivers. Two subcategories were identified under competency drivers: “Learning together” and “Informing key partners”. The subcategory “Learning together” mirrors perceptions related to competency and collaboration between nurses and social workers partnering to deliver the program. The subcategory “Informing key partners” reflects the need to inform the FCA network about the program, and questions about who should do that. Solutions included training activities to promote professional collaboration, matching support to provider experience, and planning for informing key partners.

**Table 4:** Integrated results in relation to competency drivers.

Sub-category	QUANT results			QUAL results		Integrated results
	Card sorting	Exemplar results	Summary	Exemplar excerpts	Solutions	
Learning together	Barriers = 21% Facilitators = 21% Unknowns = 58%	3 cards relating to the need for training - **barrier** for all groups. Card relating to selection of staff - **facilitator** for 4 groups.	Most competency cards (58%) were sorted as Unknowns. Unknowns were mainly related to perceptions of training and supervision. This result is understood to be because trainings had not yet taken place. The remaining cards were sorted rather evenly between alternatives Not true (Facilitators) and Completely/Partially True (Barriers).	Many strengths in competence; the group has the right competence for the task and the group feels secure. (F)	Select/hire competent staff. Assess previous experience with home visits professional teams. Let those with experience try home visits right away. Support for less experienced	***Confident & competent workforce based on previous experience (F)*** Despite the presence of unknowns and awareness of a need for training in card sorting, discussions revolved around previous professional experience working in teams and doing home visits as contributing to a sense of confidence. There was also awareness that some lacked experience and would need support.
		Card relating to unclear roles in working with the method - **barrier** for 4 groups. 6 cards relating to training and supervision quality - **unknown** for all groups.		We need descriptions of our roles, what do we do at home visits, and who does what, if possible. So that everyone gets to say what they need to say. It's important that we can cooperate. We have different knowledge; it can go wrong in the dialog with parents. (U)	Training suggestions: Team building; get-to-know-you activities; role play of home visiting situations; workshop: how do we approach (home visits) together?	***Insufficient clarity regarding professional roles during home visits (U)*** The documentation of professional roles stemmed from discussions about cards from Organizational drivers. Given the presence of many unknowns and need for training, some discussions centered around what was needed and hoped for in the training/supervision process. Clear professional roles were emphasized as critical.
Informing key partners		3 groups sorted the card, *Other colleagues know too little,* as a **barrier**		Participant 2: The politicians made the decision; it didn't come from us. (The information to partners) needs to come from the ones who assigned the task. (U) Participant 1: Midwives are key people. They need to be informed. Preschool teachers too; they’re important. Facilitator: How to go out with the information. Who? Participant 1: The operational workgroups. We’re the ones who need to formulate it. (F)	Operational workgroups develop information materials for partners and parents. Provide a training or inspiration day for co-workers/partners.	***Inform FCA partners (F)*** Card sorting and documentation reflect awareness of the need to inform the FCA network about REHV, so that prospective parents get consistent information from referral sources. While there was agreement about the need to inform and solutions, the documentation reflected that it was not clear for everyone whose responsibility it was, as demonstrated in the exemplar quote.

B, barrier; F, facilitator; S, solution; U, unknown.

#### 3.1.1 Learning together

None of the participants represented workplaces who had completed a training at the time of this study, and therefore four of the five groups perceived a lack of training and outside support as barriers. While a predominance of cards was sorted as unknowns, the cards generated group discussions about professional competence, training preferences, and suggestions and expectations for training activities. A confident and competent workforce based on previous experience was described as a facilitator. An awareness of professionals’ collective competence and confidence in working with the REHV was perceived as a facilitator. Both card sorting and field notes convey the perception that a facilitator in the implementing was that the right staff were selected to work with the program. The training was seen as more significant for team building than for training in working with families. Groups from two different districts described feeling that they already possess the competence needed and that there was an established tradition of nurses and social workers partnering in work with families. There was also awareness that some providers lacked experience and/or confidence doing home visits and working in teams and would need support.

The ones who are used to doing home visits would be able to try it [even without training] (G2).

There was a perception of insufficient clarity regarding professional roles during home visits. Most cards about the quality of training and supervision were sorted as “Unknown”. A potential barrier was described in terms of anxiety about whether or how the training offered would facilitate professionals’ ability to actualize professional collaboration during home visits. The lack of clarity as to the form for collaboration between nurses and social workers was described in connection to one of the organizational driver cards about defining roles and responsibilities, and this created concern. Groups differed in their expectations for how following the REHV method would influence the practical work with families. Some voiced a preference that training in REHV should provide a guiding framework within which there is room for flexibility; others voiced the need for clear definitions of who does what during home visits. There was a concern that insufficient articulation of roles could result in problems during home visits.

#### 3.1.2 Informing key partners

Card sorting and field notes were somewhat discrepant in relation to this topic. The card stating *Other colleagues know too little about the method* was sorted as a barrier in three of the groups. On the other hand, field notes highlighted that creating information materials and providing information to key people was something operational workgroups were able and willing to do, and therefore was classified as a facilitator. The importance of informing midwives, open playschool teachers, and parents was described. The midwives’ role was underscored; they were seen as key people due to their role in informing families about REHV.

Inform the whole midwife group. Midwives are the road into everything, need to understand their importance and their roll (G4).

Suggestions for how to inform key partners were proposed in some of the group discussions. Although there was agreement among the groups about the need to develop materials for FCA partners, one of the groups grappled with uncertainty about who was responsible for informing FCA partners, stemming from the top-down nature of the decision to implement the REHV. Some thought the politicians who made the decision to implement REHV should inform partners, others felt it should be the operational workgroups. Table 4 includes an excerpt from dialog in one group leading to agreement that the members of the operational workgroups should be the ones providing the information.

### 3.2 Organizational drivers

Table 5 depicts a summary of the integrated findings in relation to Organizational drivers, consisting of three subcategories: “Competing demands”, “Process supporter role”, and “Fidelity monitoring and follow-up evaluation”. Overall, commitment was felt to be high but potential barriers were also substantial. The subcategory “Competing demands” describes the interplay of potentially competing organizational priorities on groups’ perceived ability to plan for a high-quality implementation as conveyed both through the card sorting and in field notes. Roles and responsibilities were perceived as being inadequately defined, as exemplified in the subcategory “Process supporter role”. The subcategory “Fidelity monitoring and outcome evaluation” features concerns about how burdensome the routines for follow-up evaluations would be on personnel and families. A critique described by participants as important for front line staff was that the definition of the purpose and goals for implementing the method on a local level was inadequate. Solutions related to organizational drivers included clarification of key roles and developing procedures for evaluation tailored to local needs and resources.

**Table 5:** Integrated results related to organizational drivers.

Sub-category	QUANT results			QUAL results		Integrated results
	Card sorting	Exemplar results	Summary	Exemplar excerpts	Solutions	
Competing demands	Barriers = 35% Facilitators = 58% Unknowns = 7%	5 cards relating to support and clear prioritization of REHV coming from management were sorted by all groups as **facilitators**, and 2 more cards relating to management's commitment were rated as **facilitators** by 4 groups.	Most cards related to organizational drivers (58%) were perceived as facilitators. One third of the cards were perceived to be barriers. Despite the early phase of the implementation, few cards were sorted as unknowns. Compared to the competency and leadership drivers, the barriers are more substantial, reflecting the experience of organizational drivers as the least secure aspect of the implementation.	(The results are primarily from card sorting).		***Organizational commitment and clear priorities (F)*** Card sorting indicates the perception that there is commitment in the organization for the implementation. This includes resources, prioritization, and support from higher-ups.
		Competing demands sorted by 3 groups as a **barrier.**		Participant 1: Keep in mind that this is only 1/3 of all the children we see. We have to have other processes going at the same time. Participant 2: Yes, but don't start a bunch of new things with new collaborators. We need balance with what we have going. (B)	Don't start new partnerships or trainings during REHV implementation.	***Many other processes going on (B)*** Card sorting and field notes are consistent in illustrating that participants perceive that there is a risk of taking on too much at the same time REHV is being implemented.
		Too many methods (2 groups) and other methods competing for resources (2 groups) perceived as **barriers.**		A lot of what we do today could be refined and integrated…everyone working in the same direction (F)	We need to coordinate our parenting programs	***Common thread with other parenting programs (F)*** Although the card sorting resulted in the perception of too many or competing methods as a barrier, field notes illustrate that there is a need for the REHV method and potential for refining and coordinating parenting programs so that rather than compete, they complement each other.
Process supporter role		Four of the groups sorted the card, *It has happened that colleagues have been sent to training in the process supporter role without really understanding what will be expected of them*, as **unknown**.		Clarity about the process supporters, both in the operative group and in relation to staff. If you’re going to have a new role, it must be super clear. (B)	Appoint a process supporter. Define expectations for process supporters in relation to coworkers and managers. Articulate role descriptions, who to turn to for what. Provide training	***Process supporter role (B)*** Unclear roles and responsibilities was a barrier in card sorting results. Field notes validate this; a barrier to proceeding with pre-implementation was that the role of process supporter had not yet been clearly defined by the central implementation team.
Fidelity monitoring and outcome evaluation		We often miss following up, we need to be better at following up, we lack systematic follow-up all sorted as **barriers.**		They want a simple method for fidelity monitoring; not too time consuming or something that gets skipped due to high stress (U)	Develop procedures that are not too burdensome (for practitioners and families). Possible data sources: 2.5-year check-up at child health clinics; Session satisfaction rating scale Plan regular updates about how the follow-up evaluation is going.	***Follow up routines often lacking in practice (U)*** Card sorting resulted in classification as a barrier. There was awareness follow up is often overlooked in both social services and child health clinics. But groups were aware that these follow up routines were needed and important but not yet in place (unknown). There was a perceived concern about time demands and stress for professionals who were already stretched thin.
		3 groups perceived articulation of long-term goals as a **barrier**.		They want a way that is manageable and answers OUR questions. This is a sticking point for them. They need to find their own purpose. (B)	Each area/workplace tailor/define their own vision. Tailor evaluation to local context, needs, questions.	***Insufficient tailoring of vision, purpose, and goals to local context (B)*** Although the method was seen as a given and there was a lot of support for the implementation, there were some strong criticisms conveyed as well. A “sticking point” for front-line workers was that the purpose and goals the program were not sufficiently processed and articulated at the local level.

B, barrier; F, facilitator; REHV, Rinkeby extended home visiting; S, solution; U, unknown.

#### 3.2.1 Competing demands

The card sorting results reflect groups’ perception that support, commitment, and clear prioritization of REHV coming from higher up in the organization were facilitators. Although ten cards about organizational support were sorted as facilitators, the field notes were more centered around potential barriers. Awareness of how competing demands within the organization could create implementation barriers was evidenced by the card *Many things compete that make it hard to follow through with the implementation* being sorted as a barrier*.* Group discussions about prioritizing the REHV implementation revealed a concern about starting other new initiatives and programs at the same time, with different collaborators, and a need to focus on the REHV implementation.

We need to remind ourselves not to do a bunch of new things at the same time, just focus on the home visiting while it’s new (G3).

There was content in the field notes in response to the card *Maybe we have too many methods that we are focusing on.* One group disagreed, stating that the preventive arm of social services did not yet have any similar program or intervention. Through a reflective discussion, one group came up with a solution for how to clarify how the work with implementing REHV could complement, rather than compete with, their work with other parenting programs, and how what they were already doing could be refined and integrated. In two other groups, a solution was offered and stated in their action plan: ‘We need to coordinate our parenting programs’.

#### 3.2.2 Process supporter role

The role of process supporter, in contrast to the other leadership roles (manager and FCA coordinator; see Figure 1 above) did not exist previously but rather was developed specifically for the REHV implementation in Gothenburg. Four of the groups sorted the card, *It has happened that colleagues have been sent to training in the process supporter role without really understanding what will be expected of them,* as an unknown. The process supporter role was described as a top-down creation, so groups did not feel that they had ownership over defining that role based on local needs or context; rather they were waiting to receive information from the central implementation support team. Not knowing what process supporters would be expected to do, in relation to their colleagues and to management, was a barrier to preparing for the implementation. Groups identified an implementation barrier related to the process supporter role as evidenced by the following excerpt:

It isn’t clear for the managers what the process supporters are supposed to do. The Center needs to be clearer. Where do I turn for what? We need the frame to see what we need to go over (G3).

Groups suggested that the people assigned to this role receive an introductory training to learn what will be expected of them.

#### 3.2.3 Fidelity monitoring and outcome evaluation

The importance of planning for the outcome evaluation was discussed as involving two different needs, one relating to how much time and energy that will be required, and the other related to the purpose and goals of the initiative and targets for outcomes evaluation. While the participants understood that the procedures for outcome evaluation were yet unknowns, uncertainty was expressed regarding how burdensome the evaluation procedures would be and who would be responsible for which activities, which were described as potential sources of stress. Four groups sorted as a barrier and one group as an unknown two of the cards related to follow-up evaluation: “*We need to be better at following up and monitoring fidelity of the method”,* and “*We lack systematic follow-up”.* There was awareness of a general lack of methods for following up work with families, as evidenced by sorting *Sometimes we miss following up the methods we work with* as a barrier in four of the groups. It was perceived as difficult to follow up the work with families used because of the lack of structured systems.

It’s hard to follow up different methods that are used today within social services, but even within child health clinics, where there isn’t any structured system either (G5).

The groups understood the value of fidelity monitoring, of evaluating how well the method works, and whether it benefits families. One group expressed a need for a simple system for fidelity monitoring that would not be too time consuming. Another group described as a barrier the feeling among staff that attention to vision and goal setting had been neglected. One group underscored the need to measure how well the method works in a district that is the least similar to Rinkeby demographically. A suggestion was to use the same parent satisfaction rating system in REHV that was previously implemented in social services.

They need to see that what they do has a purpose. They need to find their own purpose, both in general and specific to them. It’s not the same for [our group] as it was in Rinkeby (G3).

### 3.3 Leadership drivers

Table 6 presents integrated findings in relation to leadership drivers, with two sub-categories: “Inner and outer context” and “Mobilizing operational workgroups”. “Inner and outer context” reflects factors identified as crucial to leadership drivers both within and outside the organization. “Mobilizing operational workgroups” mirrors more concrete leadership work needed to be taken care of before the installation phase of the implementation, like resource distribution, budget, and planning. Solutions associated with leadership drivers included proactive plans for redistribution of resources to enable long-term commitment and support for staff.

**Table 6:** Summary of integrated results in relation to leadership drivers.

Sub-category	QUANT results			QUAL results		Integrated results
	Card sorting	Exemplar results	Summary	Exemplar excerpts	Solutions	
Inner and outer context	Barriers = 20% Facilitators = 63% Unknowns = 17%	3 cards referring to getting professionals on board and the method fitting our way of working seen as **facilitators** by all groups.	Of the three categories of implementation drivers, leadership drivers had the highest proportion of cards sorted **facilitators**. Potential threats to the implementation were sorted as unknowns and **barriers.**	It's a new way of working but everyone involved is on board and very positive.	Selection of program viewed as appropriate. (F)	***Professionals’ willingness and engagement (F)*** Card sorting and field notes reflect that buy-in and positivity from professionals were clear facilitators.
		Cards with statements about urgent situations, high levels of stress and competing demands were rated as **barriers** or **unknowns**. 3 groups sorted a card about difficulty adapting the method to their workplace a **barrier.**		All it takes is a fly to make us lose focus. It's not difficult to adapt the program to our workplace in general but the model needs to be flexible for things that can happen, for example system crashes and organizational changes.	Flexible system to withstand urgencies and system crashes. Long term commitment and support in the organization (U)	***Things we cannot control (U)*** Card sorting reflects awareness that a lot can happen in an organization, like restructuring and system crashes, that could pose a threat to the implementation. Field notes convey awareness that long-term commitment and investment is needed within the entire organization.
Mobilizing operational workgroups		(Results stem primarily from qualitative content analysis)		Need for time to secure pre-implementation supports evident in field notes.	Redistribution of resources to cover work of staff assigned to REHV. Concrete planning: Budget, office space, hiring, scheduling system (B)	***Lack of protected time for operational workgroups’ planning (B)*** Field notes illustrate that it can be a challenge for members of operational workgroups to find time to meet to secure the implementation supports.

B, barrier; F, facilitator; S, solution; U, unknown.

#### 3.3.1 Inner and outer context

The operational workgroups perceived that staff desire and commitment to implementing the REHV program were facilitators. Positive attitudes about the change were evident in the card sorting and the qualitative data, manifested as the perception of staff engagement as a facilitator and a motivator for implementation and of the belief that all staff involved was “on board” and positive to the change. Few barriers were voiced in relation to professionals’ willingness and motivation to work with the REHV program. Discussions centered around unknowns that were potential barriers and difficult to predict or plan for.

Staff engagement is a facilitator, it creates motivation for the implementation (G1).

Some potential barriers were attributed to factors outside of the operational workgroups’ control. While the groups perceived the method as appropriate for their workplaces, two groups classified the card, *It can be difficult to adapt the method to our workplace*, as a barrier. The participants did not perceive the program to be difficult to adapt to their workplace in general, but believed the model needed to be flexible for things that could happen, such as system crashes, accidents, or organizational changes.

Prioritizing among individual colleagues can also be a determining factor. It’s important that the entire organization has a long-term perspective to make it sustainable (G5).

Thus, even with a great deal of staff buy-in and most leadership cards sorted as facilitators, the group discussions highlighted awareness that the facilitators might not be enough to sustain the implementation in the absence of support and flexibility in the entire organization.

#### 3.3.2 Mobilization of the operational workgroups

There were no results in the card sorting related to the concrete work of the operational workgroups other than those related to the process supporter role; rather, the content analysis of discussions illustrated that groups perceived that they needed more time together to manage their tasks. Some operational workgroups were mobilized to begin planning during the workshop because they had not yet had time to sit together to plan. The discussions identified many logistical issues and challenges that preoccupied members of the operational groups, such as how to set up and manage a booking system that could be accessible to providers working in different sectors or agencies. Another issue had to do with how to calculate what percentage of a part-time employee's position should be devoted to home visiting. One of the units lacked office space, another needed to hire staff, while others expressed concern that the budget was determined one year at a time despite families being invited to participate for 15 months. One of the groups scheduled a day and time to meet for further planning of the points that came to light during the workshop.

## 4 Discussion

### 4.1 Main findings

This article contributes knowledge about preparing for a city-wide cross-sectoral implementation of an extended home visiting program. To our knowledge, few studies have specifically focused on identifying facilitators and barriers in the preparation phase from the perspective of operational workgroups. Although several factors were considered too soon to know in the preparation phase of the implementation, some critical facilitators and barriers were identified. Main facilitators were the existence of a competent and confident workforce, and the predominantly enthusiastic expectations by staff committed to implementing the program, which was perceived as possible to integrate with existing methods. Potential barriers included unclear professional roles and responsibilities and insufficient staff and organizational capability to meet internal and external demands competing with the program and the implementation. Also, there was concern about securing necessary prerequisites for the start-up of the program, like informing key partners and setting up fidelity monitoring and evaluation routines that would not be too burdensome for the staff. Solutions were generated in response to identified facilitators, barriers, and unknowns in each category of implementation drivers.

### 4.2 Findings in relation to previous research

Previous research highlights the importance of assessing provider-related characteristics to facilitate competency drivers, such as knowledge and skills, attitudes about working evidence-based, and individual provider personality styles (41). The subcategory “Learning together” highlights the importance of assessing provider experience and level of comfort in the preparation phase, both with doing home visits and working in collaboration with another professional. Even though REHV in Gothenburg extended the previously established cross-sectoral FCA collaboration, our results indicate that different contexts possess different levels of experience and expectations for professional collaboration. A core competency component in the implementation of REHV is called the braiding method: “In the dialog, a braiding is created between our expertise and parents’ knowledge and questions. Braiding is the primary professional tool in the home visits” (42). In the evaluation of the initial development of REHV, researchers reported a concern among professionals about how the nurse–social worker partnership would form and develop given that their work with families are grounded in different traditions (12). In some groups, the participants felt that home visits could begin even without formal training, whereas others expressed a strong need for guidance with roles during home visits and a concern that the collaboration could go wrong and possibly be harmful to families. Successful collaboration rests in part on the assumption that different professionals merging expertise and relying on each other will lead to better results than what they could achieve individually (43). At the same time, common dilemmas can emerge in inter-professional partnerships related to professional identity, power, territory, and expertise (44). Integrating new research findings about professional roles during REHV into pre-implementation support, training and supervision protocols could be a way of addressing potential competency driver barriers in new implementations of REHV. For example, Barboza et al. (45) describe essential contributions of the family support social worker's role in REHV for program delivery. Inclusion of these findings in in pre-implementation training and supervision can offset perceived barriers stemming from unclear professional roles.

The need for collaboration with key FCA partners also emerged as a key concern. Establishing networks is critical in the implementation of early childhood parenting programs (18, 25). Some of the workgroups identified a lack of planning for involving FCA partners as a barrier. Discussions led to awareness of a need in implementation planning, and the participants were able to generate ideas about good ways to involve midwives, open playschool teachers, and parents. At the same time, it was not evident that the responsibility should fall on operational workgroups to inform key partners. This reflects an uncertainty and potential barrier that can emerge in the preparation phase, in particular when decisions about leadership roles and responsibilities are formed by higher-level decision makers; a barrier that is closely related to the one about unclear responsibilities categorized as an organizational driver. A lesson learned in our study is that having clearly delegated roles and responsibilities can reduce operational workgroups’ sense of uncertainty and perception of barriers in the preparation phase.

Leadership drivers include consensus building and clear articulation of how the proposed change aligns with agency mission, values, and philosophy (20), which, according to Aarons et al. (46), are considerations that should be addressed already in the exploration phase. Our results show that operational workgroups perceived commitment and enthusiasm for REHV to be facilitators, and that the program was seen as a good fit in their communities. Beyond commitment, organizational drivers during the preparation phase also encompass the perceived efficacy to implement the change (27). Organizational drivers represented the greatest number of perceived implementation barriers for the operational workgroups in this study. For example, our findings regarding concerns about having enough time and juggling different demands were similar to those found during the original REHV implementation in Rinkeby (12). For one of the operational workgroups participating in our study, the workshop session itself was the first opportunity they had to come together and begin to engage in preparatory activities, suggesting a deficit in time allotted to the operational workgroups for planning. The need for clear prioritization of REHV during the implementation period and synchronization with other parenting programs were examples of supports important for organizational capacity that operational workgroups identified as having the potential to be either facilitators or barriers, but it was too soon to know.

A facilitative administration has been proposed by Fixsen et al. (20) as an organizational driver that encompasses policies and procedures being in place to support the implementation, including staffing, space, protected time, and technical supports. The operational workgroups in our study were accountable for much of the concrete work in securing a facilitative administration for the REHV implementation in Gothenburg. The primary organizational barriers already evident in the preparation phase had to do with uncertainty about roles and responsibilities and decision support systems. Importantly, the participants described as a “sticking point” for front-line workers that they had not been involved in processing the program purpose and goals themselves. Front-line workers want to be involved in thinking about how the program fits with their traditions and ways of working, and they want their questions to be answered in the program evaluation. This finding is consistent with an important aspect raised by Yosafzai et al. (47) regarding the fundamental principle of acknowledging end-users as partners to be engaged in the process when implementing nurturing care programs. If front-line staff are not involved from the outset, there is a risk that the perceptions of barriers in the implementation will be heightened.

Given the uncertainty inherent to the preparation phase of implementation, especially regarding organizational drivers, preexisting organizational culture and climate can serve an important protective function (48). The “braiding” of knowledge inherent to the REHV program can be conceptualized as a good model for relationships between decision makers and different levels of leadership within the organization. Previous research has described the positive cascade effects of good relationships within organizational contexts in which early childhood programs are implemented (49). Collaboration and partnerships are essential for attaining SDGs, as evidenced in SDG 17, *Partnerships for the goals*. A fourth category of implementation drivers not included in Fixsen's model but proposed in other research, termed relational drivers (50), refers to factors that can create a sense of psychological safety in the midst of organizational change. In the home-visiting literature, it is well-established that the practitioner–supervisor relationship is central to program success (51). Examples of relationship-focused factors contributing to work with organizational change are mutual respect, trust, authentic relating, listening, management of conflict, and empowerment (49, 52). Assessing relational drivers as an indicator of adaptive capacity within organizations is an important task in the preparation phase, both in primary care practice (52) and in community-based implementation of early childhood programs (49). Our results suggest that attention to relational driver supports can contribute to a sense of security for operational workgroups in the preparation phase of implementing REHV which in turn can facilitate tolerance for the dynamic process of learning by doing, a feature of many successful cross-sectoral SDG initiatives (53, 54).

### 4.3 Methodological considerations

A strength of this study is the mixed methods design integrating quantitative and qualitative findings, which allowed us to get a more complete understanding of the implementation determinants perceived by the operational workgroups and their thoughts about solutions. The integration of the two datasets validates the findings and constitutes a form of data triangulation enhancing the study's credibility and trustworthiness (38). Integration also enables comparison with an emerging literature in the field using different research approaches to understanding operational workgroups’ perspectives. Another strength is the use of systematic procedures to collect and analyse data and detailed and transparent reporting of those procedures, which enhances credibility, dependability, and transferability of the findings (39). These measures may help the reader judge whether the study findings could be transferred to other, similar, contexts, e.g., other community settings in which similar nurturing care or other early childhood development initiatives are implemented. Our sample was similar to those in other published studies in Sweden looking at REHV in terms of gender and average years of experience which strengthens transferability of the findings (12, 55). Trustworthiness is further enhanced through supporting the narrative with illustrative excerpts from the different groups.

This study is not without methodological limitations. A major limitation of the study is the reliance on written notes rather than audio or video recordings, which limits the richness of the data and reduces credibility and trustworthiness of the study. A challenge to study rigor was that this research had dual purposes related to both securing implementation quality and evaluating the implementation process. The procedure was therefore designed to accommodate a real-world situation, rather than the other way around, where the research design dictates procedural decisions. One of the researchers, the first author, had dual roles in this process, entailing a risk of compromising research quality. However, embedding research into practical situations, where researchers and participants generate new knowledge together, might also enhance implementation quality (56). To protect against potential threats to trustworthiness due to researcher bias, the last author, who was neither familiar with nor had a role in the implementation of REHV, analyzed the qualitative data independently. Another step that was taken to bolster trustworthiness was the contribution of participant checking (57) by the third author, who also participated in the workshops.

A potential limitation is the use of IMPLEMENTATION DECK, given that the tool has not been validated or used in research before. We selected the tool for its potential to enhance the overall implementation by assessing and evoking reflection about facilitators and barriers related to implementation drivers and generating motivation and readiness for change among operational workgroups. Both the researchers and participants perceived the use of IMPLEMENTATION DECK as a meaningful and helpful tool for structuring group discussions that contributed to an understanding of what needed to be done to succeed with the implementation of REHV. Many questions were, however, not perceived as relevant in the current phase of implementation and the participants expressed a wish to go through the card sorting game later in the process. Thus, use of the tool as pre- and post-measurement of an implementation can be a direction for future research. Our study highlights the potential value of the tool for both research and in work with securing high-quality implementations in real-world settings.

### 4.4 Conclusions and future directions

This study expands the understanding of important implementation determinants perceived by workgroups who are planning to implement REHV, an extended home visiting program that has gained popularity in Sweden in recent years. Although several facilitators were identified in the preparation phase, such as motivation and competence within the staff, the barriers revealed may be of greater importance both to informing solutions and to providing important knowledge for future implementations of REHV and other home visiting programs as well as for other implementation endeavors in general. The barriers identified in this study, along with the associated solutions that were generated, were to a great extent centered around facilitation of collaborative processes. Findings highlight the importance of relational attributes within organizations and between the different partners in a cross-sectoral collaboration to facilitate work with implementation drivers in the preparation phase. The study contributes valuable findings to the field of implementation research and practice in early childhood development. Several aspects of our study adhere to the recommendations given by leaders in the field (17), including a need for research that pays careful attention to early-stage implementation, a need for practical guides for assessing implementation at different stages, and a need for research reporting on the use of novel methods and mixed methods. It also provides useful knowledge for decision makers and organizations preparing for cross-sectoral implementation of REHV and similar early childhood parenting programs in communities striving to attain sustainable development goals. A future direction for research is to examine how perceptions in pre-implementation relate to later outcomes and sustainability. Future studies could include using IMPLEMENTATION DECK to map implementation determinants in other implementation endeavors, as well as repeating the measure to monitor changes and emerging needs during later stages of an implementation.

## Data Availability

The raw data supporting the conclusions of this article will be made available by the authors, without undue reservation.
